# TSPY is a cancer testis antigen expressed in human hepatocellular carcinoma

**DOI:** 10.1038/sj.bjc.6602716

**Published:** 2005-08-02

**Authors:** Y-H Yin, Y-Y Li, H Qiao, H-C Wang, X-A Yang, H-G Zhang, X-W Pang, Y Zhang, W-F Chen

**Affiliations:** 1 Immunology Department, Peking University Health Science Center, Beijing 100083, China

**Keywords:** testis-specific protein Y-encoded, hepatocellular carcinoma, CT antigen, antibody response, immunotherapy

## Abstract

In search for genes associated with hepatocellular carcinoma (HCC) by cDNA microarray, we found that the transcription of TSPY, ‘testis-specific protein Y-encoded’, was upregulated in HCC. Investigation of a broad spectrum of normal and malignant tissues by RT–PCR revealed the TSPY transcript selectively expressed in normal testis, different histological types of human neoplastic tissues, and tumour cell lines. The expression of TSPY in cancer cells was further confirmed by *in situ* hybridisation. Indirect immunofluorescence microscopy analysis showed that TSPY was localised mainly in the cytoplasm of transiently transfected cells. Testis-specific protein Y-encoded was detected in 50% (16 of 32) of well- and moderately differentiated HCC patients, in 16% (four of 25) of poorly differentiated HCC patients, and in 5% (one of 19) of renal cell cancer patients. A serological survey revealed that 6.6% (seven of 106) HCC patients had anti-TSPY antibody response, demonstrating the immunogenicity of TSPY in humans. In conclusion, these data suggest that TSPY is a novel cancer/testis (CT) antigen and may be a potential candidate in vaccine strategy for immunotherapy in HCC patients.

Hepatocellular carcinoma (HCC), the major type of primary liver cancer, is one of the most prevalent cancers worldwide, and is markedly increasing in incidence because of the dissemination of hepatitis B and C virus infection (Llovet *et al*, 2003). Despite the remarkable advances in diagnostic and therapeutic techniques, prognosis of HCC still remains extremely poor ranking as the third highest cause of cancer death (Butterfield and Ribas, 2002). Therefore, the innovative and potentially disease-specific therapies for HCC are of great concern. Antigen-specific immunotherapy, as an alternative approach for treatment of HCC, is attractive due to the exquisite specificity of the immune response. Recent reports indicated that a subset of immunotherapy trials for HCC had shown clinical effects (Iwashita *et al*, 2003; Butterfield, 2004; Kuang *et al*, 2004). A prerequisite for a broad application of antigen-specific immunotherapy is the identification of appropriate target antigens capable of inducing an immune response. The discovery of cancer/testis (CT) antigens, which express in a broad spectrum of neoplasms but not in normal tissues except in testis and placenta, has provided new perspectives for antigen-specific immunotherapy (Scanlan *et al*, 2002). To date, 44 CT gene families have been identified, and the protein products of 19 gene families have been demonstrated to elicit immune responses in human (Scanlan *et al*, 2004). The immunogenicity and restricted expression of CT antigens make them ideal candidates for specific cancer immunity. However, the known CT antigens are expressed in only a fraction of cases of a given tumour type, and the progressive loss of tumour antigens is due to the immunoselection in the course of vaccination, the identification of additional CT antigens is urgently needed for developing polyvalent tumour vaccines to improve the efficacy of immunotherapy (Kalos, 2003).

Recent development of cDNA microarray technology has opened a new era in medical sciences. Several studies demonstrated the usefulness of this technique for identifying novel cancer-related genes and for disclosing genetic mechanisms in cancers (Okabe *et al*, 2001; Iizuka *et al*, 2002; Smith *et al*, 2003). We have performed cDNA microarray analysis for mining differentially expressed genes during the progress of HCC in an attempt to provide clues for identifying new therapeutic targets (Shi *et al*, 2005). In this study, we identified *TSPY*, ‘testis-specific protein Y-encoded’, which was overexpressed in human HCC, as based on cDNA microarray data. We examined *TSPY* mRNA expression in tumours of different histological types and the seroreactivity against TSPY in HCC patients, suggesting that TSPY is a novel CT antigen capable of eliciting antibody response in HCC patients. Testis-specific protein Y-encoded may provide a novel therapeutic target for immunotherapy in HCC patients.

## MATERIALS AND METHODS

### Tissues, sera, and cell lines

Tumour tissues, paired noncancerous tissues, and serum samples were obtained with informed consent from patients who underwent surgical resection at the 2nd School of Clinical Medicine, Peking University Health Science Center, China. All the samples are from male patients. Tissues destined for RNA extraction were kept frozen in liquid nitrogen immediately after separation. Tissue samples for *in situ* hybridisation were fixed in 4% formalin solution and embedded in paraffin. Serum samples were stored at −70°C freezer.

Hepatocellular carcinoma cell lines (HLE: nondifferentiated, derived from a 68-year-old male patient; Hep3B: well differentiated (WD), derived from 8-year-old male patient) and COS7 cells were obtained from Shanghai Institute of Cell Biology, Chinese Academy of Sciences (Shanghai, People's Republic of China). The other six moderately to poorly differentiated (PD) HCC cell lines of male origin, Hep-hcc-1, Hep-hcc-2, Hep-hcc-3, Hep-hcc-4, Hep-hcc-5, and Hep-hcc-6, are the gifts kindly given by Professor Ya-Jun Guo, the Second Military Medical University, China. The cDNA of melanoma (derived from male), lung (derived from male), breast, pancreas, colon (derived from female), prostate, and ovary cell lines were purchased from Clontech Laboratories Inc. (Palo Alto, CA, USA).

### RT–PCR

The expression pattern of *TSPY* transcript was determined by RT–PCR. In all, 16 different normal tissue cDNA preparations, including heart (pooled from three male Caucasians), brain (pooled from two male Caucasians), placenta, lung (pooled from two female Caucasians), liver (pooled from three male Caucasians), skeletal muscle (pooled from eight male/female Caucasians), kidney (pooled from five male/female Caucasians), pancreas (pooled from 15 male/female Caucasians), spleen (pooled from three male/female Caucasians), thymus (pooled from 18 male/female Caucasians), prostate, testis, ovary, small intestine (pooled from 32 male/female Caucasians), colon (pooled from 20 male/female Caucasians), and peripheral blood leucocyte (pooled from 550 male/female Caucasians), were purchased from Clontech Laboratories Inc. (Palo Alto, CA, USA). RNA samples extracted from tumour tissues, paired adjacent nontumour tissues, and cell lines were reversely transcribed with advantage reverse transcriptase (Clontech, Palo Alto, CA, USA). PCR primers specific for amplifying *TSPY* transcript were: forward, 5′-CAGGGCTTCTCATTCCACTC-3′; and reverse, 5′-CCATCATATTCAACTCAACAACTGG-3′. PCR was performed with 32 cycles of 20 s at 94°C, 20 s at 58°C, and 20 s at 72°C, followed by 7 min at 72°C. The amplified products were analysed on 2% agarose/Tris-acetate-EDTA gels stained with ethidium bromide. The integrity and quantity of the cDNA were evaluated by amplification of glyceraldehyde-3-phosphate dehydrogenase (*G3PDH*).

### *In situ* hybridisation

Sense and antisense probes were synthesised using T7 or SP6 with a DIG labelling kit (Roche Diagnostics, Switzerland) to generate *TSPY*-labelled riboprobes. The tissue sections (5 *μ*m) were deparaffinised, rehydrated, and incubated in 0.2 M HCl for 20 min. After washing with PBS, the tissues were treated with proteinase K at a concentration of 20 *μ*g ml^−1^ for 15 min at 37°C. After fixation with 4% paraformaldehyde for 5 min and washing in PBS, the sections were prehybridised for 1 h at 50°C in a buffer containing 50% (v v^−1^) formamide, 4 × SSC, 2 × Denhardts solution, and 250 *μ*g ml^−1^ RNA. Hybridisation was performed overnight at the same temperature in 50% (v v^−1^) formamide, 4 × SSC, 2 × Denhardts solution, 500 *μ*g ml^−1^ RNA, 10% dextran sulphate, and 2 *μ*g ml^−1^ DIG-labelled probes. Excess probes were removed by washing with 2 × SSC containing 50% formamide followed by RNase (250 *μ*g ml^−1^) treatment at 37°C for 30 min. Tissues were washed at 37°C in 2 × SSC and 0.2 × SSC. Then, the sections were incubated with alkaline phosphatase (AP)-conjugated anti-DIG Ab (Roche). Colour development was processed with nitroblue tetrazolium and 5-bromo-4-chloro-3-indolyl phosphate.

### Cell transfection and immunofluorescence

For localisation studies, we constructed the expression plasmid pcDNA-TSPY-FLAG by cloning a fragment containing the full-length ORF minus the termination codon in the *Hind*III–*Bam*HI sites of pcDNA-FLAG vector. The in-frame junction was confirmed by sequencing. COS7 cells were transfected with either vector alone as a control or vector with *TSPY* insert, using LipofectAMINE 2000 (Invitrogen, CA, USA), following the manufacturer's instructions. After incubation at 37°C for 24 h, cells were fixed with precooled 100% methanol at −20°C for 15 min. The fixed cells were blocked with 1% nonfat milk in PBS for 1 h and stained with anti-FLAG M2 mouse monoclonal antibody (mAb) (Sigma, USA) for 1 h at room temperature (RT), followed by incubation with TRITC (tetramethylrhodamine isothiocyanate)-conjugated anti-mouse immunoglobulin G antibody (IgG Ab) for 1 h at RT, and then cell nuclei were stained with Hoechst33342 for 10 min at 37°C. Images were obtained using a fluorescence microscope equipped with a Charge Couple Device camera.

### Western blot analysis

At 24 h after transfection, cultured cells were lysed in 2 × SDS sample buffer (0.1 M Tris-HCl, pH 6.8/0.2 M DTT/4% SDS/0.2% bromophenol blue/20% glycerol), and then separated by 12.5% SDS–polyacrylamide gel electrophoresis, followed by transfer to nitrocellulose membranes. After blocking in TNT solution containing 5% nonfat milk, the membrane was incubated with anti-FLAG mAb (Sigma) at RT for 1 h, followed by incubation with a horseradish peroxidase-linked goat anti-mouse IgG at RT for 1 h. Colour development was performed through incubation with 3,3′-diaminobenzidine tetrahydrochloride in 0.03% H_2_O_2_ and 50 mM Tris-HCl, pH 7.4.

### Seroreactivity analysis of TSPY

To analyse the presence of anti-TSPY Ab in HCC patient's sera, the full-length *TSPY* cDNA was cloned in the expression vector pET28a (+), and recombinant TSPY protein was produced in *Escherichia* coli with the induction of 1 mM IPTG at 42°C for 6 h. After purification by Ni^2+^ affinity chromatography, the recombinant TSPY protein fused with 6 × His tag was separated on 12.5% SDS–polyacrylamide gel electrophoresis, followed by transfer to nitrocellulose membrane. After blocking in TNT solution containing 5% nonfat milk, the membranes were incubated with sera from HCC patients at a 1 : 100 dilution for 1.5 h, and then with AP-conjugated goat anti-human IgG (Promega, USA). Serum IgG reactivity was detected with the AP substrate, 4-nitroblue tetrazolium chloride/5-bromo-4-chloro-3-indolylphosphate. Anti-His antibody (Qiagen) at a 1 : 5000 dilution was used for positive control.

## RESULTS

### Expression of *TSPY* mRNA in normal and tumour tissues

**Figure 1:**
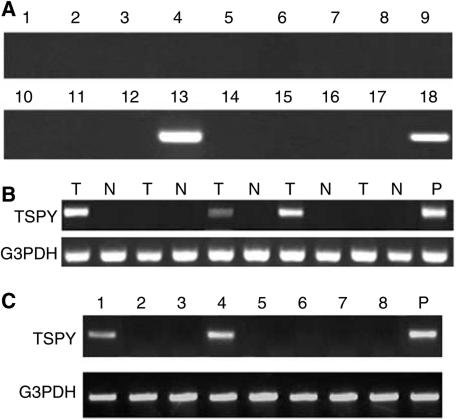
Expression of *TSPY* mRNA in normal and malignant tissues. Testis-specific protein Y-encoded mRNA expression was analysed by RT–PCR. (**A**) *TSPY* mRNA was expressed only in normal testis but not in other tissues. Lane 1: brain; 2: heart; 3: kidney; 4: liver; 5: lung; 6: pancreas; 7: skeletal muscle; 8: placenta; 9: ovary; 10: thymus; 11: prostate; 12: spleen; 13: testis; 14: small intestine; 15: colon; 16: leucocytes; 17: negative control; 18: positive control (testis as a positive control). (**B**) Expression of *TSPY* mRNA in HCC tissues. Representative expression pattern is shown in some HCC samples; testis mRNA was used as a positive control. RT–PCR for G3PDH was used to monitor the quality of the RNA sample. (T: cancerous tissues; N: adjacent noncancerous tissues; P: positive control). (**C**) Expression of *TSPY* mRNA in HCC cell lines. Lane 1: hep-hcc-1; lane 2: hep-hcc-2; lane 3: hep-hcc-3; lane 4: hep-hcc-4; lane 5: hep-hcc-5; lane 6: hep-hcc-6; lane 7: HLE; lane 8: Hep3B; P: positive control (testis as a positive control). RT–PCR for *G3PDH* was used to monitor the quality of the RNA sample.

**Figure 2:**
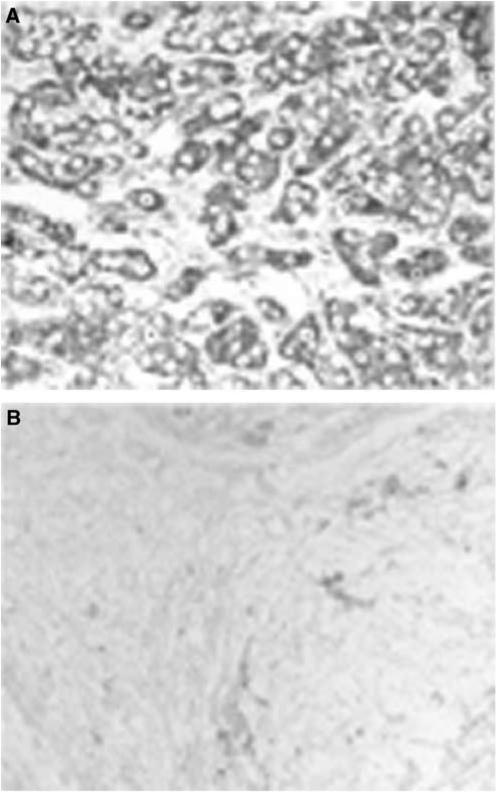
*In situ* hybridisation of *TSPY* mRNA. *In situ* hybridizstion was performed on human HCC tissues with an antisense or sense oligonucleotide probe against TSPY mRNA. (**A**) HCC tissue with antisense probe. (**B**) HCC tissue with sense probe as a control.

To examine the distribution of *TSPY* gene expression, RT–PCR was performed using cDNA reversed from mRNA of normal tissues, tumours, and cancer cell lines. Testis-specific protein Y-encoded mRNA was restricted to testis and was not detected in the various normal tissues tested, including liver, spleen, prostate, pancreas, ovary, colon, small intestine, heart, lung, peripheral blood leucocyte, brain, kidney, placenta, skeletal muscle, and thymus (Figure 1A). As the lung cDNA obtained from Clontech was of female origin, TSPY expression was further measured in normal lung tissues we collected from male patients and there was no detectable *TSPY* transcript. In addition, a number of tumours of various histological types expressed *TSPY* (Table 1). The TSPY was expressed in 35.1% (20 of 57) of HCC samples (Figure 1B). A less frequent expression was detected in renal cell cancer (one of 19). However, no expression was found in gastric cancer, lung cancer, bladder cancer and leukaemia samples. Histopathological diagnosis showed that, of the 57 HCC samples, 15 were WD, 17 were moderately differentiated (MD), and 25 were PD. The positive frequency of *TSPY* mRNA was 50% (16 of 32) in WD and MD, and 16% (four of 25) in PD HCC tissue samples. The expression of *TSPY* mRNA was in the HCC cells as demonstrated by *in situ* hybridisation analysis (Figure 2).

Of the eight HCC cell lines tested (HLE, Hep3B, hep-hcc-1, hep-hcc-2, hep-hcc-3, hep-hcc-4, hep-hcc-5, hep-hcc-6), *TSPY* mRNA was detected in hep-hcc-1 and hep-hcc-4 cell lines (Figure 1C). Among other nine cancer cell lines tested, including melanoma, ME235; lung, LX-1 and G1-117; breast, G1-101; pancreas, G1-103; colon, CX-1 and G1-112; prostate, PC3; and ovary, G1-102, TSPY was only expressed in melanoma cell line ME235.

### Subcellular localisation of TSPY

**Figure 3:**
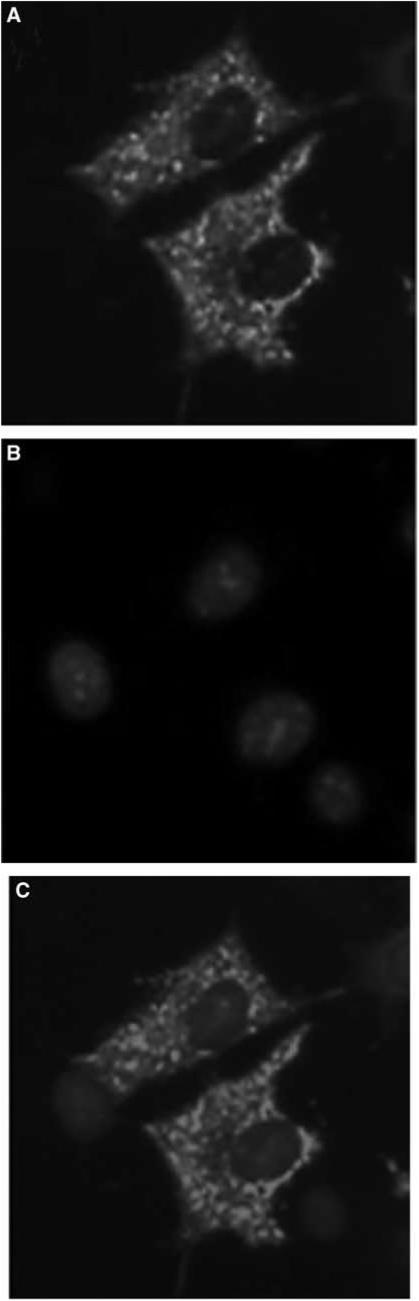
Subcellular localisation of TSPY protein. COS7 cells were transfected with pcDNA-TSPY-FLAG for 24 h, the expressed protein was then detected. (**A**) TSPY protein was mostly distributed in the cytoplasm as detected by immunofluorescence. (**B**) Nuclei stained with Hoechst33342. (**C**) Merged images of (**A**) and (**B**).

To determine the subcellular localisation of TSPY, indirect immunofluorescence for tagged TSPY was performed. FLAG-tagged *TSPY* expression plasmid was transfected into COS7 cells. Indirect immunofluorescence assay with anti-FLAG Ab demonstrated that FLAG-tagged TSPY was mainly in the cytoplasm of transfected COS7 cells (Figure 3). Testis-specific protein Y-encoded protein was also detected by Western blot in COS7 cells transiently transfected with pcDNA-TSPY-FLAG plasmid using anti-FLAG Ab, the molecular weight is approximately 48 kDa.

### Survey of anti-TSPY seroreactivity in HCC patients

**Figure 4:**
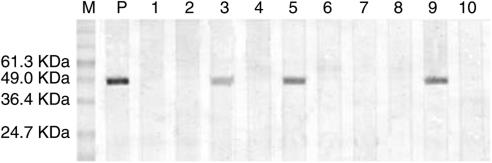
Serological reactivity of the recombinant TSPY protein in HCC patients. The antibody in serum samples was assessed by Western blot assay. M: protein marker; P: positive control; lanes 1 and 2: negative controls with normal sera; lanes 3, 5, and 9: positive reaction with sera from HCC patients; lanes 4, 6–8, and 10: negative reaction with sera from HCC patients.

To get an impression of the frequency of anti-TSPY antibodies, the sera of 106 HCC patients and 46 healthy controls were tested for Ab reactivity against recombinant TSPY by Western blot (Figure 4). Seven HCC patients had serum Ab responses specific to recombinant TSPY protein (6.6%), whereas all the healthy controls were negative.

## DISCUSSION

A prerequisite for a broad application of antigen-based vaccine for immunotherapy is the identification of a wide spectrum of immunogenic tumour antigens expressed predominately in human cancers (Kalos, 2003; Scanlan *et al*, 2004). Cancer/testis antigens are attractive targets in immunotherapy for their limited expression in normal tissue of testis, an immunological privileged site unable to provoke immune responses. To date, 44 CT gene families have been identified; however, the protein products of only three gene families have been found to induce coordinated humoral and cellular responses, including NY-ESO-1, MAGE-A, and SSX antigens (Scanlan *et al*, 2004). Some preliminary results have been reported for immunotherapy with MAGE-derived peptides (Marchand *et al*, 1995; Toungouz *et al*, 2001) and NY-ESO-1 peptide or protein (Jager *et al*, 2000; Chen *et al*, 2004; Davis *et al*, 2004). In this study, we have demonstrated that TSPY is a novel CT antigen.

Our examination of *TSPY* mRNA expression pattern has confirmed previous reports (Zhang *et al*, 1992; Schnieders *et al*, 1996) that human *TSPY* was not expressed in normal tissues except for testis. Importantly, we found the *TSPY* transcript was selectively expressed in different histological human tumours and tumour cell lines with the dominant expression in HCC. Since *TSPY* mRNA was reported to be sequence diversity, direct sequencing of cDNAs prepared from HCC and testicular tissues has been performed. Testis-specific protein Y-encoded transcript from HCC was found to be identical to that from testis, and both belong to *TSPY* major type encoding a protein of 308 amino acids as identified by Schnieders *et al* (1996). The expression profile of *TSPY* in normal tissues, tumour samples and tumour cell lines has categorised this gene in the group of CT antigens. Testis-specific protein Y-encoded differs from the known CT antigens by its chromosomal localisation. Most of the hitherto known CT antigens have been mapped to the X chromosome with the exception of a few CT antigens that have been mapped to autosome (Scanlan *et al*, 2002; Lee *et al*, 2003; Loriot *et al*, 2003). Testis-specific protein Y-encoded gene was located on human proximal Yp, within the GBY (gonadoblastoma locus on the Y chromosome) critical region (Lau *et al*, 2000). Since HCC is three to four times more often in men than women (Kew, 2002; El-Serag, 2004), the relatively high frequency of *TSPY* in HCC patients might be of significance in aetiology and therapeutics.

The biological function of most CT antigens is unclear except for a few CT antigens, such as SCP-1, which is involved in meiotic chromosome pairing (Tureci *et al*, 1998); OY-TES-1, involved in the packaging of acrosin in the sperm head (Baba *et al*, 1994); CT15/fertilin *β*, involved in egg/sperm membrane interactions (Vidaeus *et al*, 1997; Primakoff and Myles, 2000); SSX, reported to have a transcription-repressor function (Brett *et al*, 1997). Testis-specific protein Y-encoded is evolutionarily conserved on the mammalian Y chromosome, its homologous gene families have been found in great apes (Kim and Takenaka, 1996), cattle (Vogel *et al*, 1997), monkey (Kim *et al*, 1997), and other mammal (Lau, 1999). These suggest that *TSPY* might be an important gene acting in male germ cell generation. Testis-specific protein Y-encoded has been indeed reported to function in early spermatogenesis, immediately prior to the spermatogonia to spermatocyte transition (Schnieders *et al*, 1996). Detailed database and sequence analysis suggest that TSPY belongs to a superprotein family, which includes the proto-oncogene *SET* and the nucleosome-assembly factor *NAP-1*, both interact specifically with mitotic cyclin B and are suggested to play a role in DNA replication (Schnieders *et al*, 1996). SET has been identified as a potent inhibitor of protein phosphatase 2A, a major mammalian protein serine threonine phosphatase that regulates diverse cellular processes (Li *et al*, 1996). Recently, *SET* was found to be an inhibitor of tumour suppressor gene *NM-23H1* (Fan *et al*, 2003), and involved in Galpha(12)-mediated signalling pathways (Kumar *et al*, 2004). In addition, NAP-1 is needed in a transcriptional activation in cooperation with p300 (Asahara *et al*, 2002). Advances in the characterisation of SET/NAP functions may provide some important clues to the biological role of the *TSPY* gene not only in spermatogenesis but also in hepatocarcinogenesis.

In this study, the expression of *TSPY* mRNA in HCC was relevant to tumour differentiation status. Testis-specific protein Y-encoded was expressed at high proportion (50%) in WD HCC and relatively small proportion (16%) in PD HCC. It is well known that the HCC often shows dedifferentiation from WD HCC to PD HCC during multistep progression (Mise *et al*, 1998; Sakamoto *et al*, 1991). The different expression frequencies of *TSPY* transcript in WD and PD, together with the structure relationship of TSPY to SET and NAP, suggests that TSPY possibly involve in hepatocarcinogenesis and reversely involve in progression of HCC.

A serological survey has shown that seven of 106 HCC patients exhibited spontaneous humoral immune response to TSPY. Although the frequency of Ab response in HCC patients is low as most of the other CT antigens, the TSPY *in vivo* is immunogenic. Further investigation on the TSPY-mediated T-cell immune response is in process. The restricted expression in tumours and testis and its immunogenicity imply that TSPY is a potential vaccine candidate for immunotherapy in HCC.

## Figures and Tables

**Table 1:** Expression of *TSPY* transcript in malignant tissues

**Tumour type**	**TSPY expression, no. positive/no. tested**
HCC	20/57
Renal cell carcinoma	1/19
Lung carcinoma NSCLC	0/13
Bladder cancer	0/8
Lymphoma or leukaemia	0/11
Gastric carcinoma	0/10

TSPY=testis-specific protein Y-encoded; HCC=hepatocellular carcinoma; NSCLC=non-small-cell lung carcinoma
